# HIV treatment engagement in the context of COVID-19: an observational global sample of transgender and nonbinary people living with HIV

**DOI:** 10.1186/s12889-021-10977-5

**Published:** 2021-05-12

**Authors:** Arjee Javellana Restar, Henri M. Garrison-Desany, Tyler Adamson, Chase Childress, Gregorio Millett, Brooke A. Jarrett, Sean Howell, Jennifer L. Glick, S. Wilson Beckham, Stefan Baral

**Affiliations:** 1 Department of Epidemiology, Johns Hopkins School of Public Health, 615 N Wolfe St, Baltimore, MD 21205 USA; 2 Department of Health, Policy, and Management, Johns Hopkins School of Public Health, Baltimore, MD USA; 3 School of Law and School of Criminology and Criminal Justice, Northeastern University, Boston, MA USA; 4 amfAR, The Foundation of AIDS Research, Washington, DC USA; 5 Hornet, San Francisco, CA USA; 6 Department of Health, Behavior, and Society, Johns Hopkins School of Public Health, Baltimore, MD USA; 7 Department of International Health, Johns Hopkins School of Public Health, Baltimore, MD USA

**Keywords:** Coronavirus, COVID-19, Transgender people living with HIV, HIV

## Abstract

**Background:**

HIV services, like many medical services, have been disrupted by the COVID-19 pandemic. However, there are limited data on the impacts of the COVID-19 pandemic on HIV treatment engagement outcomes among transgender (trans) and nonbinary people. This study addresses a pressing knowledge gap and is important in its global scope, its use of technology for recruitment, and focus on transgender people living with HIV. The objective of this study is to examine correlates of HIV infection and HIV treatment engagement outcomes (i.e., currently on ART, having an HIV provider, having access to HIV treatment without burden, and remote refills) since the COVID-19 pandemic began.

**Methods:**

We utilized observational data from the Global COVID-19 Disparities Survey 2020, an online study that globally sampled trans and nonbinary people (*n* = 902) between April and August 2020. We conducted a series of multivariable logistic regressions with lasso selection to explore correlates of HIV treatment engagement outcomes in the context of COVID-19.

**Results:**

Of the 120 (13.3%) trans and nonbinary people living with HIV in this survey, the majority (85.8%) were currently on HIV treatment. A smaller proportion (69.2%) reported having access to an HIV provider since COVID-19 control measures were implemented. Less than half reported being able to access treatment without burdens related to COVID-19 (48.3%) and having the ability to remotely refill HIV prescription (44.2%). After adjusting for gender in the multivariable models, younger age and anticipated job loss were significantly associated with not having access to HIV treatment without burden. Outcomes also significantly varied by geographic region, with respondents reporting less access to an HIV provider in nearly every region outside of South-East Asia.

**Conclusion:**

Our results suggest that currently taking ART, having access to an HIV provider, and being able to access HIV treatment without burden and remotely refill HIV medication are suboptimal among trans and nonbinary people living with HIV across the world. Strengthening support for HIV programs that are well-connected to trans and nonbinary communities, increasing remote access to HIV providers and prescription refills, and providing socioeconomic support could significantly improve HIV engagement in trans and nonbinary communities.

**Supplementary Information:**

The online version contains supplementary material available at 10.1186/s12889-021-10977-5.

## Introduction

The novel coronavirus disease (COVID-19) continues to ravage communities around the world, impacting all areas of life. As of April 24, 2021, SARS-CoV-2 has infected over 137 million people and led to nearly 2.9 million deaths [1]. To mitigate the impact, countries have relied on a range of measures, including closures of schools and businesses, limits on gatherings, border closures, and lockdowns [2]. These measures have unintentionally upended healthcare systems, leading to reductions in access to care, cancellations of elective/ non-emergency surgeries, and rising levels of adverse mental health conditions [3–5]. Of particular concern are impacts to already marginalized communities and an exacerbation of existing health inequities. A growing body of research highlights the barriers to care that transgender (trans) and nonbinary individuals experience as a result of stigma, discrimination, and minority stress [6–8]. Yet there remains a gap in knowledge about how these barriers have evolved within the context of COVID-19.

Trans and nonbinary individuals already face heightened barriers to care, such as a limited number of providers, widespread transphobia among staff, or lack of adequate provider-focused training in working with trans and nonbinary individuals [9–11]. Furthermore, such experiences of care have a direct impact on prescribed treatment, adherence, and impacts to mental health, particularly within the context of HIV and among trans and nonbinary people living with HIV [12–14]. Additionally, the health needs of trans and nonbinary individuals are often forgotten or neglected in disaster relief responses, which can lead to an increase in negative, population-level health outcomes [15].

Structural barriers, coupled with the unprecedented nature of the COVID-19 pandemic, will likely leave this community uniquely vulnerable to disrupted engagement in HIV treatment and requires a targeted and tailored approach to address such vulnerability going forward [4, 5, 16]. Given the evolving and unknown nature of this virus, there have been coordinated efforts to prioritize individuals with preexisting conditions, compromised immune systems, and high risk of illness or death [3, 17–19]. However, previous research has shown that amid similar disasters, there are also interruptions to HIV care and engagement [20]. While there has been a proliferation of research on the ramifications of COVID-19 among those living with HIV [3, 5], little, if any, has been dedicated to documenting engagement in HIV treatment among trans and nonbinary individuals in the context of COVID-19. This is despite the widespread acknowledgment that globally, trans and nonbinary people are considered a key population in HIV mitigation and are particularly vulnerable to loss of care [21, 22].

In order to mitigate the impacts of COVID-19 on HIV treatment engagement for trans and nonbinary individuals, it is imperative to characterize and understand the extent to which access to HIV treatment has been impacted. Drawing on lessons learned from the HIV epidemic [23], this study sought to characterize the impact of COVID-19 on HIV treatment engagement among trans and nonbinary individuals living with HIV around the world. We also examined differences in HIV status, and treatment and engagement-related outcomes (e.g., currently on ART, having an HIV provider, having access to HIV treatment without burden, and access to remote refills) across geographic regions, socioeconomic status, and employment.

## Methods

### Study sample, procedures, and design

For best-practice reporting of cross-sectional study design, we utilized the STROBE checklist (Supplemental, Supporting Checklist).

This is a secondary analysis of data from the Global COVID-19 Disparities Survey 2020, a study that examined the impact of the COVID-19 pandemic on trans and nonbinary wellbeing among social networking applications (apps) members. This study specifically focuses on trans and nonbinary people’s HIV status and treatment and engagement-related outcomes such as currently on ART, having an HIV provider, having access to HIV treatment without burden, and remote refills. Study procedures have been reported elsewhere [24]. In brief, participants were recruited online via “Hornet” and “Her” social networking/dating apps. Survey invitations were sent out to app members who used their apps in the past year. Participants were screened for eligibility and were eligible if they are: (1) at least 18 years old, (2) an app member, and (3) provided electronic consent.

We utilized best-practice approaches to maximize data quality and reduce information bias, including a deduplication process via (1) removal of any non-unique IP addresses, and (2) a cross-matching process that flags identical responses to 20 random variables. Other approaches included removing participants with incomplete responses (i.e., completed less than 90% of the survey), response time below the piloted time of 7 mins, illogical responses, and those with no HIV status data.

### Ethics

The Johns Hopkins School of Public Health Institutional Review Board reviewed and approved this study (IRB #00000287). All enrolled participants provided electronic informed consent and consent for publication.

### Measures and analyses

We included variables in this analysis based on previous research [9–11, 22] and within the parameters of measures that were available in the secondary dataset (Supplemental, Survey Questionnaire). We compared demographics (i.e., gender identity, age, education, socioeconomic status, migrant status, region of residence according to the World Health Organization (WHO) definition [25], urban/rural setting, and racial/ethnic minority status) and socio-economic loss due to COVID-19 pandemic (e.g., anticipated income reduction, anticipated insurance loss, anticipated job loss/unemployment, cutting meals) by HIV status. We then compared demographics and socioeconomic loss due to the COVID-19 pandemic among HIV-positive trans and nonbinary people who were currently on ART, had an HIV provider, had access to HIV treatment without burden, and had remote refills since the COVID-19 pandemic began.

Bivariate analyses were conducted using *χ* ^2^ or Fisher’s Exact tests appropriately to describe relationships of the independent variables, stratified by HIV status. A series of multivariable regressions was used to build each model per outcome (e.g., currently on ART, having an HIV provider, having access to HIV treatment without burden, and remote refills). Given the exploratory nature of this study, prior to analysis, we utilized lasso to select the key variables to include in the model [26]. All of the variables that were selected by the lasso procedure were included per model. Additionally, given our modest sample size, we utilized a nonparametric bootstrapping procedure with 1000 iterations to strengthen our confidence intervals and reduce Type 1 error for each of our model [27]. Following guidelines from Restar and colleagues [28], we utilized a gender-inclusive approach to the analysis – that is, given that no significant differences were found between HIV status and gender, we did not analyze each model by gender in separate analysis but instead analyzed the entire sample while controlling for gender identity in all of the adjusted multivariable models. Lastly, we noted which variables were considered to be significant at *p* < 0.05. All analyses were performed using StataSE version 16.1.

## Results

### Sample characteristics

Initially, the survey had 4031 responses which, following data quality processing, resulted in an overall sample of 3139. For this analysis, we further limited our sample to only participants who identified as transgender or nonbinary, resulting in a final sample of 902 participants. Table 1 shows an overview of demographic and socioeconomic characteristics of the sample by HIV status. Overall, a total of 64.4% of trans and nonbinary people in this sample reported their HIV status as negative, 13.3% as positive, and 21.3% as unknown. Among those who reported their HIV status as positive (*n* = 120), 72.5% identified as nonbinary, 4.2% trans masculine, and 23.3% trans feminine. Additionally, among nonbinary respondents who participated in the study, a total of 14.4% (87/604) reported living with HIV compared to 14.2% (5/35) trans masculine and 10.6% (28/263) trans feminine people reported living with HIV.

**Table 1:** Sample demographics and socio-economic loss impact characteristics by HIV status in a global sample of transgender and nonbinary people (*n* = 902)

	Total n (%)	HIV-negative n (%)	HIV-positive n (%)	HIV-unknown n (%)	*p*-value
Total	902 (100.00)	590 (64.41)	120 (13.30)	192 (21.29)	
**Demographics**					
Gender identity					
Nonbinary	604 (66.96)	391 (66.27)	87 (72.50)	126 (65.62)	0.642 ^f^
Trans masculine	35 (3.80)	22 (3.73)	5 (4.17)	8 (4.17)	
Trans feminine	263 (29.16)	177 (30.00)	28 (23.33)	58 (30.21)	
Age					
18–29 years old	463 (51.33)	308 (52.20)	57 (47.50)	98 (51.04)	0.558
30–39 years old	267 (29.60)	170 (28.81)	39 (32.50)	58 (30.21)	
40–29 years old	119 (13.19)	78 (13.22)	13 (10.83)	28 (14.58)	
50 or more years old	53 (5.88)	34 (5.76)	11 (9.17)	8 (4.17)	
Education					
Less than college	213 (23.83)	137 (23.30)	28 (23.73)	48 (25.53)	0.822
College/Some college	681 (76.17)	451 (76.70)	90 (76.27)	140 (74.47)	
Level of socioeconomic status					
Lower	141 (15.68)	87 (14.77)	19 (15.97)	35 (18.35)	0.010^f^
Lower-middle	445 (49.50)	277 (47.03)	69 (57.98)	99 (51.83)	
Upper-middle	272 (30.26)	195 (33.11)	23 (19.33)	54 (28.27)	
Upper	41 (4.56)	30 (5.09)	8 (6.72)	3 (1.57)	
Migrant status					
No/Unsure	754 (84.81)	482 (83.10)	104 (86.67)	168 (88.89)	0.130
Yes	135 (15.19)	98 (16.90)	16 (13.33)	21 (11.11)	
WHO continent region					
South-East Asia	217 (24.72)	124 (21.60)	36 (30.51)	57 (30.65)	< 0.001
Americas	83 (9.45)	61 (10.63)	14 (11.86)	8 (4.30)	
Eastern Mediterranean	80 (9.11)	64 (11.15)	9 (7.63)	7 (3.76)	
Africa	34 (3.87)	22 (3.83)	9 (7.63)	3 (1.61)	
Europe	424 (48.29)	277 (48.26)	48 (40.68)	99 (53.23)	
Western Pacific	40 (4.56)	26 (4.53)	2 (1.69)	12 (6.45)	
Setting					
Urban	658 (71.11)	449 (76.36)	85 (70.83)	124 (64.58)	0.005
Rural	242 (26.89)	139 (23.64)	35 (29.17)	68 (35.42)	
Racial/Ethnic Minority					
No/Not Sure	662 (73.64)	425 (72.16)	82 (68.91)	155 (81.15)	0.022
Yes	237 (26.36)	164 (27.84)	37 (31.09)	36 (18.85)	
**Socio-economic Loss due to COVID-19 Indicators**					
Income reduction (anticipated)					
No	239 (27.10)	167 (28.99)	21 (17.65)	51 (27.27)	0.040
Yes	643 (72.90)	409 (71.01)	98 (82.35)	136 (72.73)	
Insurance loss (anticipated)					
No	382 (61.51)	265 (64.79)	43 (49.43)	74 (59.20)	0.023
Yes	239 (38.49)	144 (35.21)	44 (50.57)	51 (40.80)	
Job loss/unemployment (anticipated)					
No	753 (84.23)	502 (86.11)	97 (80.83)	154 (80.63)	0.104
Yes	141 (15.77)	81 (13.89)	23 (19.17)	37 (19.37)	
Cutting Meals					
No	510 (60.36)	345 (61.61)	61 (54.46)	104 (60.12)	0.369
Yes	335 (39.64)	215 (38.39)	51 (45.54)	69 (39.88)	

^f^ Fisher Exact Test. Column percentages are reported. Sample sizes stratified by variables may not add up to total sample size due to missingness

A majority of the sample identified their gender as nonbinary (67.0%), followed by trans feminine (29.2%) and trans masculine (3.8%). Half the sample were participants between ages 18–29 years old (51.3%), had attained some college education (76.2%), and were from lower-middle (49.5%) and upper-middle (30.3%) socioeconomic status. A total of 15.2% identified as migrants. Most of the sample were from European (48.3%) and South-East Asian WHO regions (24.7%), and a majority (71.1%) were residing in urban areas. More than a quarter (26.4%) identified as racial/ethnic minorities. Additionally, 72.9% of the sample anticipated income reduction, 38.5% anticipated insurance loss, 15.8% anticipated job loss/unemployment, and 39.6% had cut meals. Level of socioeconomic status, WHO region, urban/rural setting, racial/ethnic minority, anticipated income reduction, and anticipated insurance loss were independently associated with HIV status.

### HIV treatment and access engagement

**Fig. 1:**
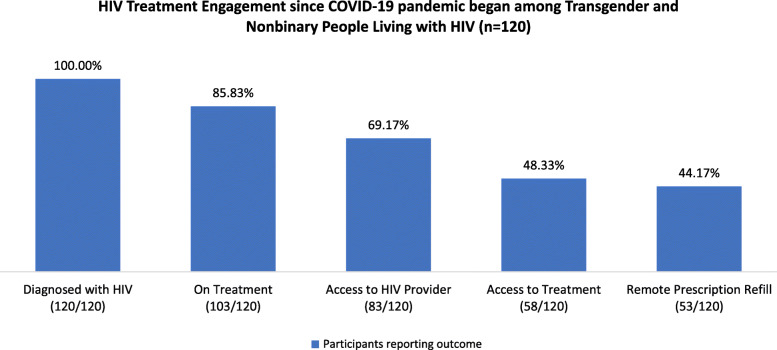
HIV treatment engagement since COVID-19 pandemic began among transgender and nonbinary people living with HIV

Figure 1 displays HIV treatment and access engagement outcomes. Among trans and nonbinary people in this sample who reported living with HIV (13.3%), the majority (85.8%) were currently on treatment. A smaller proportion (69.2%) reported having access to an HIV provider since pandemic control measures were implemented. Similarly, less than half reported being able to access treatment without burdens related to COVID-19 (48.3%) and being able to refill their prescription remotely (44.2%).

### Multivariable logistic regression analyses of HIV treatment and access engagement outcomes

Table 2 displays the results of final adjusted multivariable logistic regressions with lasso variable selection per HIV treatment engagement outcome. Specifically, the first adjusted regression model (outcome: current HIV treatment) included the following variables: gender identity, age, education, level of socioeconomic status, and region. The second adjusted regression model (outcome: access to HIV provider) included gender identity, age, region, and anticipated job loss/unemployment. The third adjusted regression model (outcome: access to treatment) included gender identity, age, education level of socioeconomic status, migrant status, region, anticipated income reduction, anticipated job loss/unemployment, and having cut meals. And lastly, the fourth adjusted regression model (outcome: remote prescription refill) included gender identity and level of socioeconomic status.

**Table 2:** Results of adjusted multivariable logistic regression with lasso variable selection: Correlates of HIV treatment engagement in the context of COVID-19 in a global sample of transgender and nonbinary people living with HIV

HIV treatment engagement outcomes	Lasso-selected factors	Adjusted OR (95% CI)	*p*-value
**On treatment**			
	Gender identity		
	Nonbinary	ref	
	Trans masculine	0.55 (0.34–0.90)*	0.018
	Trans feminine	0.88 (0.72–1.07)	0.229
	Age		
	18–29 years old	ref	
	30–39 years old	1.07 (0.92–1.23)	0.345
	40–29 years old	1.04 (0.85–1.28)	0.649
	50 or more years old	0.97 (0.68–1.37)	0.866
	Education		
	Less than college	ref	
	College/Some college	1.27 (1.02–1.59)*	0.033
	Level of socioeconomic status		
	Lower	ref	
	Lower-middle	0.90 (0.63–1.29)	0.593
	Upper-middle	0.84 (0.56–1.26)	0.413
	Upper	0.83 (0.41–1.65)	0.603
	WHO continent region		
	South-East Asia		
	Americas	1.01 (0.83–1.23)	0.854
	Eastern Mediterranean	0.90 (0.73–1.10)	0.325
	Africa	1.23 (0.98–1.53)	0.062
	Europe	0.84 (0.61–1.16)	0.300
	Western Pacific	0.85 (0.48–1.48)	0.570
**Access to HIV Provider**			
	Gender identity		
	Nonbinary	ref	
	Trans masculine	0.89 (0.46–1.72)	0.748
	Trans feminine	0.94 (0.75–1.18)	0.637
	Age		
	18–29 years old	ref	
	30–39 years old	1.19 (0.97–1.45)	0.089
	40–49 years old	1.29 (1.01–1.64)*	0.036
	50 or more years old	1.30 (1.02–1.65)*	0.031
	WHO continent region		
	South-East Asia	ref	
	Americas	0.65 (0.49–0.86)*	0.002
	Eastern Mediterranean	0.85 (0.56–1.30)	0.475
	Africa	0.65 (0.44–0.96)*	0.035
	Europe	0.78 (0.63–0.92)*	0.006
	Western Pacific	0.37 (0.29–0.46)*	< 0.001
	Job loss/unemployment (anticipated)		
	No	ref	
	Yes	1.11 (0.91–1.34)	0.292
**Access to Treatment**			
	Gender identity		
	Nonbinary	ref	
	Trans masculine	0.88 (0.60–1.30)	0.548
	Trans feminine	0.87 (0.72–1.06)	0.179
	Age		
	18–29 years old	ref	
	30–39 years old	1.36 (1.01–1.84)*	0.041
	40–29 years old	1.63 (1.17–2.26)*	0.003
	50 or more years old	1.31 (1.03–1.67)*	0.026
	Education		
	Less than college	ref	
	College/Some college	0.97 (0.86–1.08)	0.622
	Level of socioeconomic status		
	Lower	ref	
	Lower-middle	0.84 (0.71–1.01)	0.060
	Upper-middle	0.99 (0.82–1.19)	0.935
	Upper	0.64 (0.47–1.88)	0.065
	Migrant status		
	No/Unsure	ref	
	Yes	0.89 (0.72–1.09)	0.279
	WHO continent region		
	South-East Asia	ref	
	Americas	1.30 (1.09–1.55)*	0.003
	Eastern Mediterranean	0.74 (0.46–1.18)	0.217
	Africa	0.90 (0.70–1.15)	0.413
	Europe	0.99 (0.82–1.19)	0.924
	Western Pacific	0.87 (0.29–2.58)	0.803
	Income reduction (anticipated)		
	No	ref	
	Yes	1.21 (0.87–1.70)	0.247
	Job loss/unemployment (anticipated)		
	No	ref	
	Yes	0.79 (0.63–0.90)*	0.044
	Cutting Meals		
	No	ref	
	Yes	0.86 (0.71–1.05)	0.167
**Remote Prescription Refill**			
	Gender identity		
	Nonbinary	ref	
	Trans masculine	1.44 (0.96–2.16)	0.075
	Trans feminine	0.98 (0.79–1.23)	0.910
	Level of socioeconomic status		
	Lower	ref	
	Lower-middle	1.13 (0.88–1.46)	0.326
	Upper-middle	1.06 (0.77–1.46)	0.708
	Upper	1.54 (1.03–2.30)*	0.033

*p < 0.05, *OR* odds ratio, *95% CI* 95% Confidence Interval, *WHO* World Health Organization. Each model/outcome ran under a nonparametric bootstrapping procedure with 1000 iterations

In the final regression model (current HIV treatment), trans masculine people living with HIV had lower odds of being on current HIV treatment (adjusted odds ratio [aOR] = 0.55, 95% Confidence Interval [CI] = 0.34–0.90) compared to nonbinary people living with HIV. Moreover, trans and nonbinary people living with HIV who attained some college education had higher odds of being on current HIV treatment compared to those with less than college education.

Moreover, trans and nonbinary people living with HIV between ages 40–49 years old (aOR = 1.29, 95%CI = 1.01–1.64) and 50 or more years old (aOR = 1.30, 95%CI = 1.02–1.65) had higher odds of having access to an HIV provider compared to those between 18 and 29 years old. Moreover, trans and nonbinary people living with HIV in the Americas (aOR = 0.65, 95%CI = 0.49–0.86), Africa (aOR = 0.65, 95% CI = 0.44–0.96), Europe (aOR = 0.78, 95% CI = 0.63–0.92) and Western Pacific (aOR = 0.37, 95% CI = 0.29–0.46) had lower odds of accessing an HIV provider since COVID-19 pandemic control measures were implemented compared to those who are living with HIV in the South-East Asia region,

In the final regression model (access to treatment), trans and nonbinary people living with HIV between ages of 30–29 years old (aOR = 1.36, 95%CI = 1.01–1.84), 40–49 years old (aOR = 1.31, 95%CI = 1.03–1.67), and 50 or more years old (aOR = 1.63, 95%CI = 1.17–2.26), had higher odds of having access to treatment compared to those between ages 18–29 years old. Compared to those in the South-East Asia region, those residing in the Americas had higher odds of accessing HIV treatment without burdens related to COVID-19 (aOR = 1.30, 95% CI = 1.09–1.55). Moreover, those who anticipated job loss/unemployment had lower odds of accessing HIV treatment without burdens related to COVID-19 compared to those who did not (aOR = 0.79, 95% CI = 0.63–0.90).

Finally, the ability to refill HIV prescriptions remotely was significantly higher among those from upper socioeconomic status compared to those from a lower socioeconomic status (aOR = 1.54, 95%CI = 1.03–2.30).

## Discussion

The results of this observational study reflect the high burden of HIV among the trans and nonbinary community. The majority of participants identified as nonbinary, which is often an understudied portion of the transgender community [1, 2] and highlights the novelty of our results. Notably, we found the majority of the sample who anticipated job loss/unemployment had lower odds of accessing HIV treatment without burdens related to COVID-19, and that those with higher socioeconomic status had higher odds of having the ability to refill HIV prescriptions remotely. This pattern suggests significant economic precarity, as well as a general replication of the widening wealth gap among the broader population [29, 30]. Also notable was the difference in odds of accessing an HIV provider and accessing HIV treatment by geographic region since the pandemic began. While trans and nonbinary people living with HIV in South-East Asia had higher odds of accessing HIV treatment relative to those living in the Americas, Africa, Europe and the Western Pacific, those residing in the Americas had higher odds of accessing such treatment without COVID-19 burdens. Similarly, those who are older than the age group 18 to 29 years old report higher access to HIV providers and to HIV treatment, and those who attained college education are more likely to be currently on HIV treatment compared to those with less than college education. These results may partially reflect differing regional responses to the pandemic over time, while also showing that more affluent subgroups of trans and nonbinary populations (e.g., educated, from upper socioeconomic status) have been able to engage and maintain HIV treatment during the pandemic. While more research must be done to confirm these hypotheses, this study lends valuable insight into how COVID-19 restrictions have impacted access to care among this understudied and underserved population across the globe.

This study shows the importance of testing among transgender populations, and the particular nuances related to gender identity and HIV status. Roughly 20% of our study population did not know their HIV status. While this comports with prior reports of lower testing among transgender populations compared to cisgender populations [3], it is of particular concern given our sample population was recruited from a dating app and are at potentially greater risk than a population-based sample of transgender people [31]. Among those who did know their HIV positive status, a majority (72.5%) identified as nonbinary people. This further supports recommendations for tailored testing strategies to trans and nonbinary people, in order to more quickly inform individuals of their HIV status and risk.

Moreover, we found that those with higher socioeconomic status had higher odds of being able to refill their prescription remotely while those who anticipated job loss/unemployment had lower odds of accessing HIV treatment without burdens related to COVID-19, which may suggest that the disruptive effects of the pandemic may have been lessened for trans and nonbinary people with greater economic resources than others. This potentially divergent recovery period is in line with other theories of the economic impact of COVID-19 [30]. In the US, for example, there are reports of a widening wealth gap between higher income earners who were able to more seamlessly transition to remote work, while maintaining their income, jobs, and insurance coverage over the period [30]. By extension, income security likely makes it easier to maintain care, including continuing to pay for prescription or accessing care; while individuals in lower socioeconomic status who faced reduced income seemed to struggle to maintain care.

A similar difference in coping is seen geographically. Trans and nonbinary people with HIV in the Americas, Europe, Western Pacific, and Africa had lower odds of accessing a provider or treatment since pandemic control measures were implemented. Because of the cross-sectional nature of the data, it is difficult to determine if this is due to regional differences in access to care in general, or to changes in access due to the pandemic. Our study sample may have been less likely to access treatment compared to other transgender people due to prior issues preceding the pandemic [32], including lack of transgender-competent providers in their area or reluctance to disclose transgender status to providers and therefore not receiving relevant/tailored care, structural barriers such as lack of income for appointments and medications, lack of transportation to appointments or pharmacies, or experiencing discrimination at appointments. Further research must be done to understand if this reduced average is related to temporal changes in care. Moreover, given that country-level policies and socio-political climates for transgender rights and health vary within a region, it is particularly important to look into country-level analysis and/or region-specific reporting as a point of future research to understand how access to both in primary and specifically transgender care were impacted by COVID-19 among countries with various levels of social and political acceptance of transgender communities.

For instance, if our results reflect healthcare changes specific to the pandemic period, it may reflect challenges that phases of lockdown and financial hardship had on trans and nonbinary people, which differed by WHO region. Comparing the South-East Asian region to the European region and extrapolating the Oxford COVID-19 Restriction Tracker [8] to our study period of April to August, the South-East Asian region generally experienced stricter restrictions in the beginning, which eventually gave way to the same or lower levels of restrictions beginning between roughly May or July. Within the European region, there were a greater number of fluctuations in levels of restrictions, with higher levels being implemented early on, their reduction by June and July, and finally an increase in restrictions beginning in September. Differences in respective pandemic responses may have resulted in different disruptions to healthcare [9], where the stop-start nature of some country responses and poor communication about risks may have made it more difficult for trans and nonbinary people to navigate healthcare resources in these regions. In the US, which had such fluctuations, healthcare utilization through August was reduced across nearly all medical specialties, including adult primary care and immunology [10]. This remained true even for telemedicine appointments which would not be burdened by other barriers to care. Further research must be conducted to understand region-specific correlates to treatment access by region.

Other important findings worth noting include differences in HIV treatment engagement outcomes by age and gender. Specifically, we found that trans masculine people who are living with HIV are less likely, compared to nonbinary people, to report being on current HIV treatment. Moreover, younger trans and nonbinary adults were less likely to have access to HIV prevention and treatment compared to older trans and nonbinary adults. These results suggest that there are differences across age and gender identity strata and that there is a need to explore how these results may differ by subgroups globally, given that trans and nonbinary communities are a non-monolithic group [33]. More importantly, these results provide information for current HIV treatment programs operating under COVID pandemic conditions, particularly regarding which subgroups of trans and nonbinary people living with HIV are in need of bolstering outreach strategies. It is critical for HIV/AIDS treatment services to adapt sensitive and tailored trans programming strategies to subgroups of trans and nonbinary populations.

### Strengths

This study had a number of strengths, including its timeliness in the COVID-19 pandemic, and its focus on an often-underrepresented population that is likely more vulnerable to suboptimal HIV treatment engagement risk and socioeconomic burdens from these necessary governmental restrictions. Our data was collected globally from April to August 2020, during early-pandemic restrictions. Given the fast-changing nature of COVID-19 prevalence rates and the policy responses from governments, our data represents an important snapshot into how this landscape of restrictions, re-openings, and their socioeconomic and healthcare burden may have impacted trans and nonbinary people’s access to HIV treatment across the world. Additionally, our data was comprised of trans and nonbinary people sampled across the globe, particularly those living with HIV. The challenge to identify and enroll trans and nonbinary people in research has been previously described [12, 13], and the use of an app-driven sampling scheme, as opposed to sampling based in health systems or physical venues, may reduce selection bias potentially seen in prior studies [14, 15]. Finally, we assessed HIV treatment outcomes that supports delineating steps for HIV treatment engagement to continued medication adherence. In order to ensure eventual medication adherence, understanding these interim steps of care and the reduction in retention is necessary. Without access to medication, no adherence is possible.

### Limitations

This study is not without limitations. First, the cross-sectional nature of this study prevents us from inferring the directionality of the associations, as well as general understanding of changes in our outcomes over time. Second, the convenience online sampling approach via mobile apps limits the generalizability of our results and is not representative of all trans and nonbinary populations globally, especially those living with HIV who are not on these mobile apps. Third, due to the self-report responses of this survey, outcomes such as HIV status may have been underreported given that HIV is more stigmatized in some areas of the world compared to others [16]. Our study may have benefited from having biomarkers for more objective estimation. Lastly, this analysis used a nonparametric bootstrap procedure to help reduce Type 1 error inflation while making multiple comparisons. While such procedures do not guarantee control of Type 1 error, the goal of this paper was to generate hypotheses for future research and confirmation. Despite these limitations, the Global COVID-19 Disparities Survey 2020 is one of the largest surveys that sample trans and nonbinary populations globally and the survey data provided us with insights into the lives of already marginalized populations at the time of COVID-19 pandemic.

## Public health implications

Trans and nonbinary populations around the world continue to be significantly impacted by the COVID-19 pandemic, facing both HIV burden and lack of access to HIV-related treatment and care. This study was able to provide insight into how COVID-19 has impacted HIV treatment engagement in this understudied population. Moreover, given that trans and nonbinary communities bear the brunt of structural inequalities and are marginalized across intersections of racism, sexism, classism, and transphobia, it is critical for policymakers, program designers, and public health stakeholders to center trans and nonbinary populations across policies, health services programming, and COVID disaster-relief programs [34]. Access to remote HIV-related services, such as telehealth and remote prescriptions, were only partially accessible among trans and nonbinary communities across the world. Thus, innovative solutions to access treatment and providers during pandemic-related disruptions are necessary to retain vulnerable populations in care, including: (1) ensuring HIV surveillance systems include trans and nonbinary gender identities, (2) allowing more flexible remote prescription options for HIV medications, (3) mandating insurance policies to increase coverage for access to telehealth service, (4) providing gender-sensitivity and -competency trainings for providers on the frontlines of COVID-19 care, in order to reduce potential negative interactions with trans and nonbinary individuals, and (5) equitably allocating funds to support programs and the healthcare workforce providing HIV care for trans and nonbinary populations. This study provides important groundwork for further research and policies that benefit trans and nonbinary people during the COVID-19 pandemic.

## Supplementary Information

**Additional file 1.** Cross-sectional Study STROBE Checklist.

**Additional file 2.** Survey Questionnaire.

## Data Availability

The datasets used and/or analysed during the current study are available from the corresponding author on reasonable request.
