# Supplementary material for: HIV treatment engagement in the context of COVID-19: an observational global sample of transgender and nonbinary people living with HIV
Source: BMC Public Health. 2021 May 12;21:901. doi: 10.1186/s12889-021-10977-5 (PMC8114659; doi:10.1186/s12889-021-10977-5)
Supplement: Supplementary file 1 — Additional file 1. Cross-sectional Study STROBE Checklist. [file 12889_2021_10977_MOESM1_ESM.docx]

**HIV Treatment Engagement in the Context of COVID-19: An Observational Global Sample of Transgender and Nonbinary People Living with HIV**

Arjee Javellana Restar, PhD^1^*; Henri M. Garrison-Desany, MSPH^1^; Tyler Adamson, MPH^2^; Chase Childress, MS^3^; Gregorio Millett, MPH^4^; Brooke A. Jarrett, MSPH^1^; Sean Howell, BA^5^; Jennifer L. Glick, PhD^6^; S. Wilson Beckham, PhD^6,7^; Stefan Baral, MD^1^

^1^ Department of Epidemiology; Johns Hopkins School of Public Health; Baltimore, MD, USA

^2^ Department of Health, Policy, and Management; Johns Hopkins School of Public Health; Baltimore, MD, USA

^3^ School of Law and School of Criminology and Criminal Justice, Northeastern University, Boston, MA.

^4^ amfAR, The Foundation of AIDS Research, Washington, DC, USA

^5^ Hornet; San Francisco, CA, USA

^6^ Department of Health, Behavior, and Society; Johns Hopkins School of Public Health; Baltimore, MD, USA

^7^ Department of International Health; Johns Hopkins School of Public Health; Baltimore, MD, USA

***Correspondence**: Arjee Javellana Restar, PhD, MPH, Johns Hopkins School of Public Health, Department of Epidemiology, 615 N Wolfe St, Baltimore, MD 21205. Email: arestar1@jhmi.edu

**Keywords:** Coronavirus; COVID-19; transgender people living with HIV; HIV

**Supplement:** Cross-sectional Study STROBE Checklist.

|  | Item No | Recommendation | Page Numbers |
| --- | --- | --- | --- |
| **Title and abstract** | 1 | (*a*) Indicate the study’s design with a commonly used term in the title or the abstract | 1 |
|  |  | (*b*) Provide in the abstract an informative and balanced summary of what was done and what was found | 4-5 |
| Introduction | | | |
| Background/rationale | 2 | Explain the scientific background and rationale for the investigation being reported | 5-6 |
| Objectives | 3 | State specific objectives, including any prespecified hypotheses | 6 |
| Methods | | | |
| Study design | 4 | Present key elements of study design early in the paper | 6-7 |
| Setting | 5 | Describe the setting, locations, and relevant dates, including periods of recruitment, exposure, follow-up, and data collection | 7 |
| Participants | 6 | (*a*) Give the eligibility criteria, and the sources and methods of selection of participants | 7 |
| Variables | 7 | Clearly define all outcomes, exposures, predictors, potential confounders, and effect modifiers. Give diagnostic criteria, if applicable | 8 |
| Data sources/ measurement | 8* | For each variable of interest, give sources of data and details of methods of assessment (measurement). Describe comparability of assessment methods if there is more than one group | *8* |
| Bias | 9 | Describe any efforts to address potential sources of bias | 8 |
| Study size | 10 | Explain how the study size was arrived at | 8 |
| Quantitative variables | 11 | Explain how quantitative variables were handled in the analyses. If applicable, describe which groupings were chosen and why | 8 |
| Statistical methods | 12 | (*a*) Describe all statistical methods, including those used to control for confounding | 8-9 |
|  |  | (*b*) Describe any methods used to examine subgroups and interactions | 8-9 |
|  |  | (*c*) Explain how missing data were addressed | 8-9 |
|  |  | (*d*) If applicable, describe analytical methods taking account of sampling strategy | N/A |
|  |  | (*e*) Describe any sensitivity analyses | 8-9 |
| Results | | | |
| Participants | 13* | (a) Report numbers of individuals at each stage of study—eg numbers potentially eligible, examined for eligibility, confirmed eligible, included in the study, completing follow-up, and analysed | 9 |
|  |  | (b) Give reasons for non-participation at each stage | 9 |
|  |  | (c) Consider use of a flow diagram | N/A |
| Descriptive data | 14* | (a) Give characteristics of study participants (eg demographic, clinical, social) and information on exposures and potential confounders | 9, Table 2 |
|  |  | (b) Indicate number of participants with missing data for each variable of interest | 9, Table 2 |
| Outcome data | 15* | Report numbers of outcome events or summary measures | 9-10, Figure 1 |
| Main results | 16 | (*a*) Give unadjusted estimates and, if applicable, confounder-adjusted estimates and their precision (eg, 95% confidence interval). Make clear which confounders were adjusted for and why they were included | 10, Table. 3 |
|  |  | (*b*) Report category boundaries when continuous variables were categorized | 8-11 |
|  |  | (*c*) If relevant, consider translating estimates of relative risk into absolute risk for a meaningful time period | N/A |
| Other analyses | 17 | Report other analyses done—eg analyses of subgroups and interactions, and sensitivity analyses | 9-11 |
| Discussion | | | |
| Key results | 18 | Summarise key results with reference to study objectives | 11-15 |
| Limitations | 19 | Discuss limitations of the study, taking into account sources of potential bias or imprecision. Discuss both direction and magnitude of any potential bias | 15 |
| Interpretation | 20 | Give a cautious overall interpretation of results considering objectives, limitations, multiplicity of analyses, results from similar studies, and other relevant evidence | 11-15 |
| Generalisability | 21 | Discuss the generalisability (external validity) of the study results | 15-16 |
| Other information | | | |
| Funding | 22 | Give the source of funding and the role of the funders for the present study and, if applicable, for the original study on which the present article is based | N/A |
