# Supplementary material for: HIV treatment engagement in the context of COVID-19: an observational global sample of transgender and nonbinary people living with HIV
Source: BMC Public Health. 2021 May 12;21:901. doi: 10.1186/s12889-021-10977-5 (PMC8114659; doi:10.1186/s12889-021-10977-5)
Supplement: Supplementary file 2 — Additional file 2. Survey Questionnaire. [file 12889_2021_10977_MOESM2_ESM.docx]

**HIV Treatment Engagement in the Context of COVID-19: An Observational Global Sample of Transgender and Nonbinary People Living with HIV**

Arjee Javellana Restar, PhD^1^*; Henri M. Garrison-Desany, MSPH^1^; Tyler Adamson, MPH^2^; Chase Childress, MS^3^; Gregorio Millett, MPH^4^; Brooke A. Jarrett, MSPH^1^; Sean Howell, BA^5^; Jennifer L. Glick, PhD^6^; S. Wilson Beckham, PhD^6,7^; Stefan Baral, MD^1^

^1^ Department of Epidemiology; Johns Hopkins School of Public Health; Baltimore, MD, USA

^2^ Department of Health, Policy, and Management; Johns Hopkins School of Public Health; Baltimore, MD, USA

^3^ School of Law and School of Criminology and Criminal Justice, Northeastern University, Boston, MA.

^4^ amfAR, The Foundation of AIDS Research, Washington, DC, USA

^5^ Hornet; San Francisco, CA, USA

^6^ Department of Health, Behavior, and Society; Johns Hopkins School of Public Health; Baltimore, MD, USA

^7^ Department of International Health; Johns Hopkins School of Public Health; Baltimore, MD, USA

***Correspondence**: Arjee Javellana Restar, PhD, MPH, Johns Hopkins School of Public Health, Department of Epidemiology, 615 N Wolfe St, Baltimore, MD 21205. Email: arestar1@jhmi.edu

**Keywords:** Coronavirus; COVID-19; transgender people living with HIV; HIV

**Supplement:** Survey Questionnaire

**Demographics:**

| Gender | How would you define your gender identity? (choose all that apply) |
| --- | --- |
|  | Non-binary (Gender nonbinary/Gender diverse (also genderqueer, gender nonconforming, gender expansive, agender) |
|  | Trans masculine (Transgender woman (male to female) |
|  | Trans feminine (Transgender man (female to male) |
| Age | How old are you? |
|  | 18-29 years old |
|  | 30-39 years old |
|  | 40-29 years old |
|  | 50 or more years old |
| Education | How many years of education have you completed? |
|  | Less than college (Less than 6 years, Between 6 and 12 years, Trade school or vocational training) |
|  | College/Some college (Some university but no degree, University degree or more) |
| SES | What is your socioeconomic status |
|  | Upper |
|  | Upper middle |
|  | Lower middle |
|  | Lower |
| Migrant Status | Are you a migrant to the country in which you currently live? |
|  | I am |
|  | No/Unsure |
| WHO Region | In which country do you currently live? [select country] |
| Setting | Which of the following describes the place where you now live? |
|  | Urban (A capital city, A large city, A suburb near a large city) |
|  | Rural (A small city or town, A rural area or village, A farm or isolated house) |
| Racial/Ethnic Minority | Do you consider yourself a member of an ethnic or racial minority? |
|  | Yes |
|  | No |

**Socio-economic Loss due to COVID-19 Indicators**

| Income Reduction (anticipated) | How much are you expecting your income to reduce because of the COVID-19 crisis? |
| --- | --- |
|  | No income reduction (0% ) |
|  | Income reduction (1-100%) |
| Insurance Loss (anticipated) | Do you expect to lose your health insurance coverage because of the COVID-19 crisis? |
|  | Yes (Definitely yes, probably yes) |
|  | No (might or might not, probably, definitely not) |
| Job Loss /Unemployment (anticipated): | Do you expect to lose your job or be unemployed because of the COVID-19 crisis? |
|  | Yes |
|  | No/Unsure |
| Cutting Meals | Since the COVID-19 crisis began, have you had to cut the size of your meals or skip meals because there was not enough money for food? |
|  | Yes |
|  | No |

**HIV Treatment and Engagement Outcomes:**

| HIV Status | Do you know your HIV status? |
| --- | --- |
|  | I’m HIV-Negative |
|  | I’m HIV-Positive |
|  | I don’t know |
| On treatment: | If HIV-Positive, are you currently taking antiretroviral treatment (ART)/medicine for HIV? |
|  | Yes |
|  | No |
| Access to HIV provider: | Since the beginning of COVID-19 related social isolation in your country, by that or any other name, have you been able to see your HIV provider if you needed to? |
|  | Yes (in person, via telemedicine) |
|  | No (because it is closed, because of reduced hours) |
| Access to Treatment | Are you able to access your HIV medicine despite special measures related to COVID-19? |
|  | No (I cannot access my HIV medicine, or I can access my HIV medicine, but the process is burdensome or complicated) |
|  | Yes (I can access or refill my HIV medicine without much or any complication) |
| Remote Prescription Refill | Can you refill your HIV medicine prescription remotely? |
|  | Yes (my HIV provider has made it possible during the COVID-19 crisis) |
|  | No (I cannot remotely refill my HIV medicine) |
